# Recurrent Pulmonary Embolism After Pulmonary Endarterectomy During Rivaroxaban Therapy in a Resource-Limited Setting

**DOI:** 10.1016/j.jaccas.2026.109220

**Published:** 2026-07-06

**Authors:** Aniqa Batool, Muhammad Imran Ansari, Irfan Amjad Lutfi, Muhammad Mohsin

**Affiliations:** National Institute of Cardiovascular Diseases, Karachi, Pakistan

**Keywords:** anticoagulation, hyperhomocysteinemia, pulmonary embolism, pulmonary endarterectomy, resource-limited setting

## Abstract

**Background:**

Recurrent pulmonary embolism after endarterectomy despite adequate anticoagulation in high-risk patients remains a significant clinical challenge in low and middle-income countries.

**Case Summary:**

We report the case of 37-year-old man with hyperhomocysteinemia and prior pulmonary endarterectomy who presented with recurrent pulmonary embolism while on rivaroxaban. Imaging demonstrated a large thrombus in the right pulmonary artery with right ventricular dysfunction on echocardiography. In the absence of right heart catheterization, pulmonary hypertension could not be established; therefore, the case was managed as recurrent pulmonary embolism. Given high surgical risk and lack of advanced cardiopulmonary support, the patient was treated with systemic thrombolysis, inferior vena cava filter placement, anticoagulation escalation, and initiation of riociguat.

**Discussion:**

This case highlights the challenges of managing recurrent pulmonary embolism in low-resource settings and underscores the importance of individualized therapeutic strategies.

**Take-Home Message:**

Careful risk stratification and switching of anticoagulation should be considered for patients with recurrent pulmonary embolism and hyperhomocysteinemia.

## Visual Summary

**Figure:**
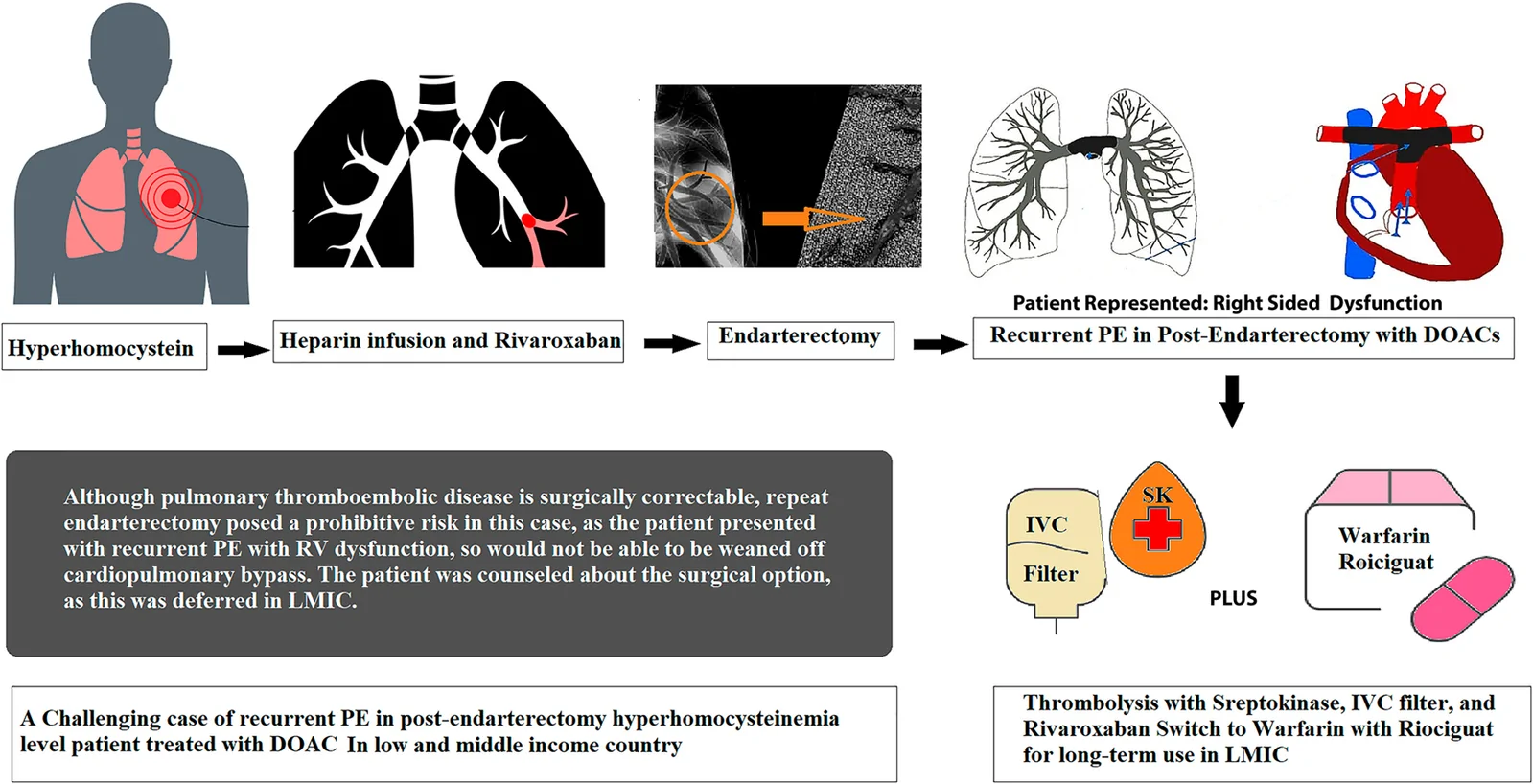

**Visual Summary:**
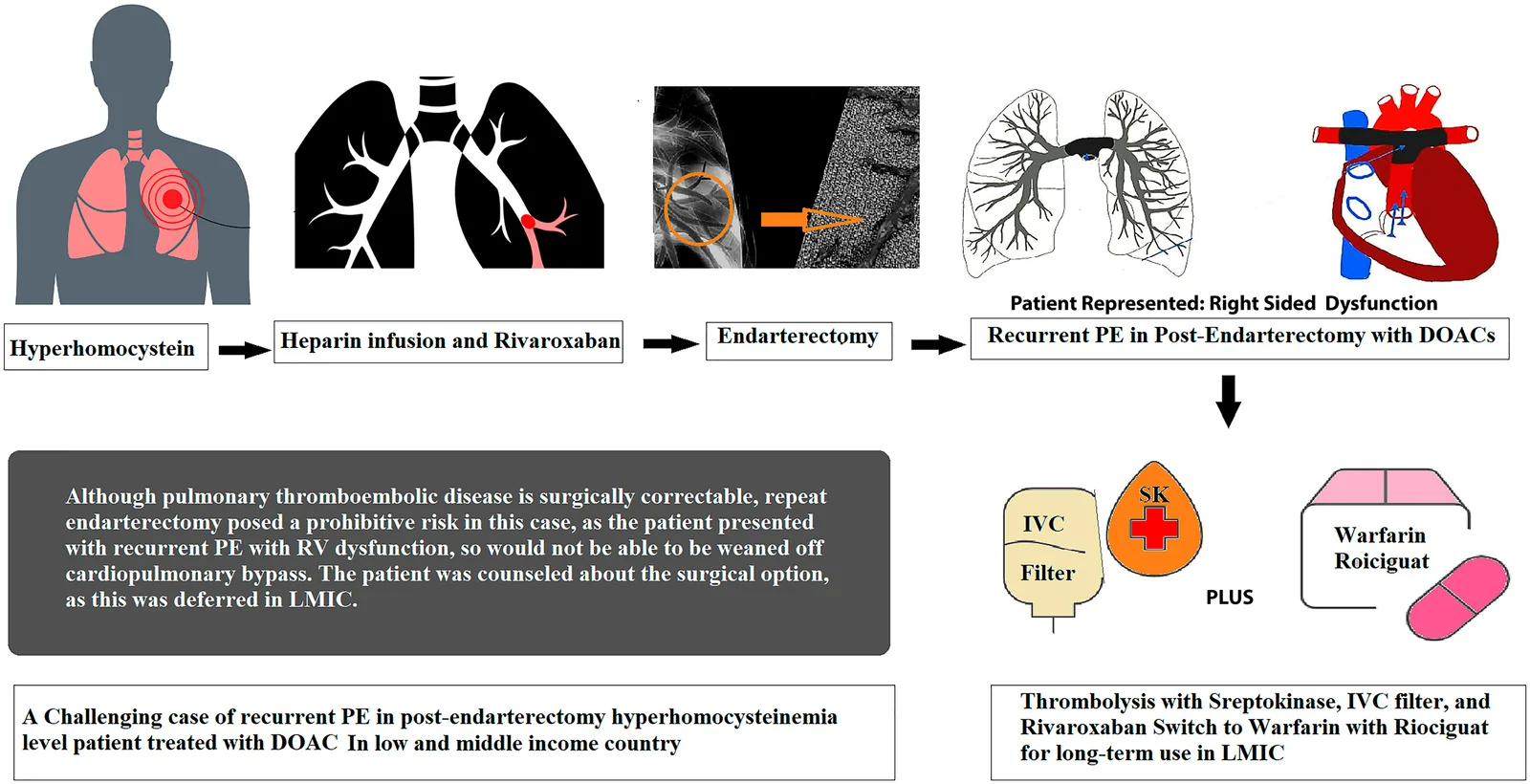
Recurrent Pulmonary Embolism Despite DOAC Therapy in a Postendarterectomy Patient With Hyperhomocysteinemia: Transition to Warfarin, IVC Filter Placement, and Initiation of Pulmonary Vasodilator Therapy in a Resource-Limited Setting DOAC = direct oral anticoagulant; IVC = inferior vena cava; LMIC = low- and middle-income countries; PE = pulmonary embolism; RV = right ventricular.

## History of Presentation

A 37-year-old man presented with worsening shortness of breath for 1 week. On examination, he was hypoxemic with oxygen saturation around 86% on room air, as well as tachycardic.

## Past Medical History

The patient had a known case of hyperhomocysteinemia (60 μmol/L) and pulmonary embolism (PE), and he had undergone pulmonary endarterectomy 5 years earlier. He had been receiving rivaroxaban 20 mg once daily, folic acid, and multivitamin supplements. Medication adherence was confirmed through a detailed patient interview, with no reported interruptions in anticoagulation therapy.

## Differential Diagnosis

The differential diagnosis included recurrent acute PE, residual chronic thromboembolic obstruction after prior pulmonary endarterectomy, chronic thromboembolic pulmonary disease, and pulmonary hypertension secondary to chronic thromboembolic disease.

In patients presenting with PE and elevated homocysteine levels, other causes of hypercoagulability should be considered. These include inherited thrombophilias such as factor V Leiden thrombophilia, protein C deficiency, protein S deficiency, and antithrombin III deficiency. Autoimmune conditions like antiphospholipid syndrome were also considered.

## Investigations

A thrombophilia work-up was completed. Hyperhomocysteinemia (60 μmol/L) was confirmed. Testing for antiphospholipid antibodies, protein C, protein S, and antithrombin III deficiency was negative. Baseline laboratory values are shown in Table 1.

**Table 1:** Baseline Laboratory Investigations

Laboratory Parameter	Value (Units)
Hemoglobin	16.7 g/dL
Hematocrit	50%
Platelet count	457 × 10^9^/L
RBC	2.93 × 10^12^/L
Creatinine	0.9 mg/dL
Magnesium	1.96 mg/dL
Phosphorus	4.1 mg/dL
Sodium	138 mmol/L
Potassium	4.0 mmol/L
Chloride	110 mmol/L
Bicarbonate	27 mmol/L
Albumin	3.7
SGPT	30 U/L
SGOT	40 U/L
ALP	56 U/L
GGT	76 U/L
Direct bilirubin	0.39 mg/dL
Total bilirubin	1.25 mg/dL
CRP	1.3 mg/L
INR	1.2
TSH	0.96 mIU/L
HBsAg	Nonreactive
HIV	Nonreactive
HCV antibody	Nonreactive
Total cholesterol	150 mg/dL
LDL cholesterol	92 mg/dL
HDL cholesterol	46 mg/dL
Triglycerides	120 mg/dL
VLDL cholesterol	30 mg/dL

ALP = alkaline phosphatase; CRP = C-reactive protein; GGT = gamma-glutamyl transferase; HBsAg = hepatitis B surface antigen; HCV = hepatitis C virus; INR = international normalized ratio; LDL = low-density lipoprotein; RBC = red blood cells; SGOT = serum glutamic-oxaloacetic transaminase; SGPT = serum glutamate pyruvate transaminase; TSH = thyroid-stimulating hormone; VLDL = very low-density lipoprotein.

**Figure 1:**
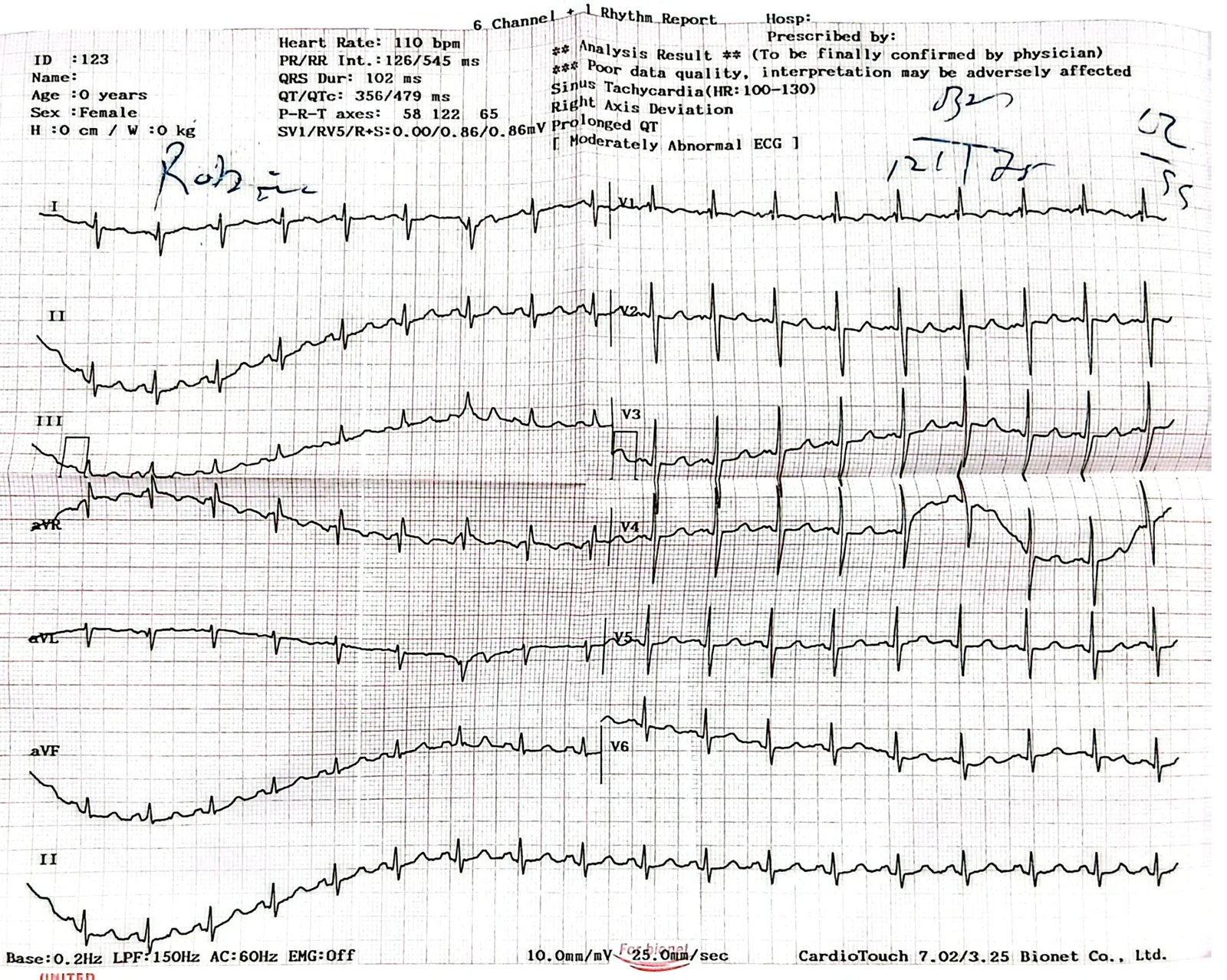
Electrocardiogram Suggestive of Pulmonary Embolism A 12-lead electrocardiogram demonstrating sinus tachycardia with features of right heart strain, including an S1Q3T3 pattern and T-wave inversions in leads V_1_ to V_3_. Findings are consistent with acute right ventricular strain in pulmonary embolism.

A 12-lead electrocardiogram demonstrating sinus tachycardia with features of right heart strain, as shown in Figure 1.

**Figure 2:**
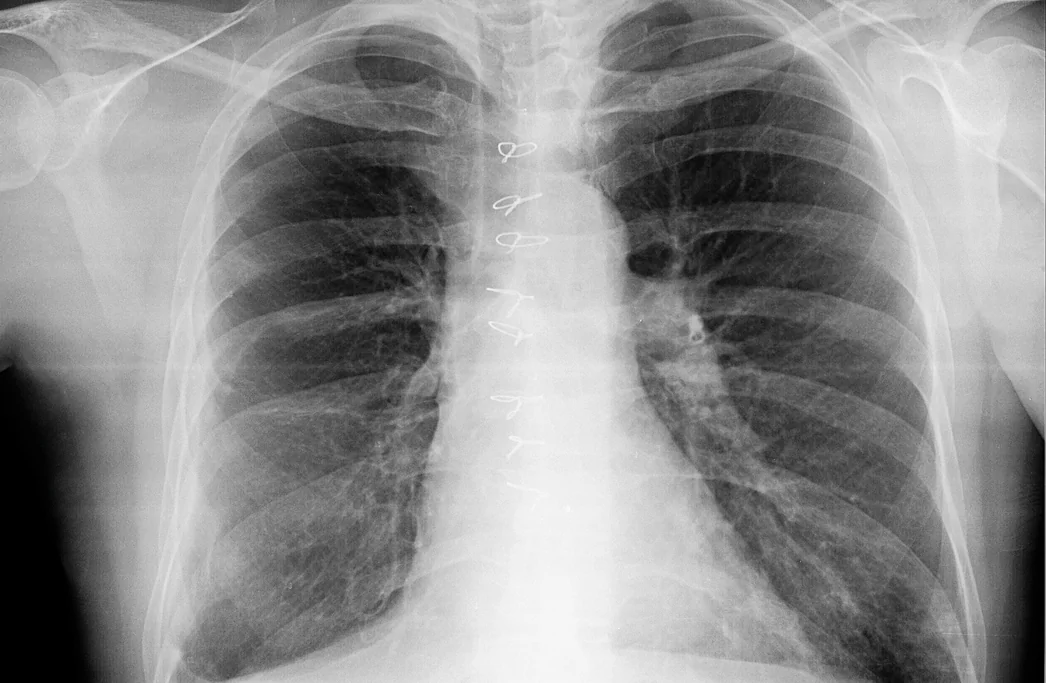
Chest Radiography Chest radiograph demonstrated poststernotomy changes consistent with prior pulmonary endarterectomy, with mild prominence of perihilar pulmonary vasculature and blunting of the right costophrenic angle suggestive of a small pleural effusion. No significant cardiomegaly was observed.

Chest radiography demonstrated poststernotomy changes consistent with prior pulmonary endarterectomy, with mild prominence of perihilar pulmonary vasculature and blunting of the right costophrenic angle suggestive of a small pleural effusion, as shown in Figure 2.

**Figure 3:**
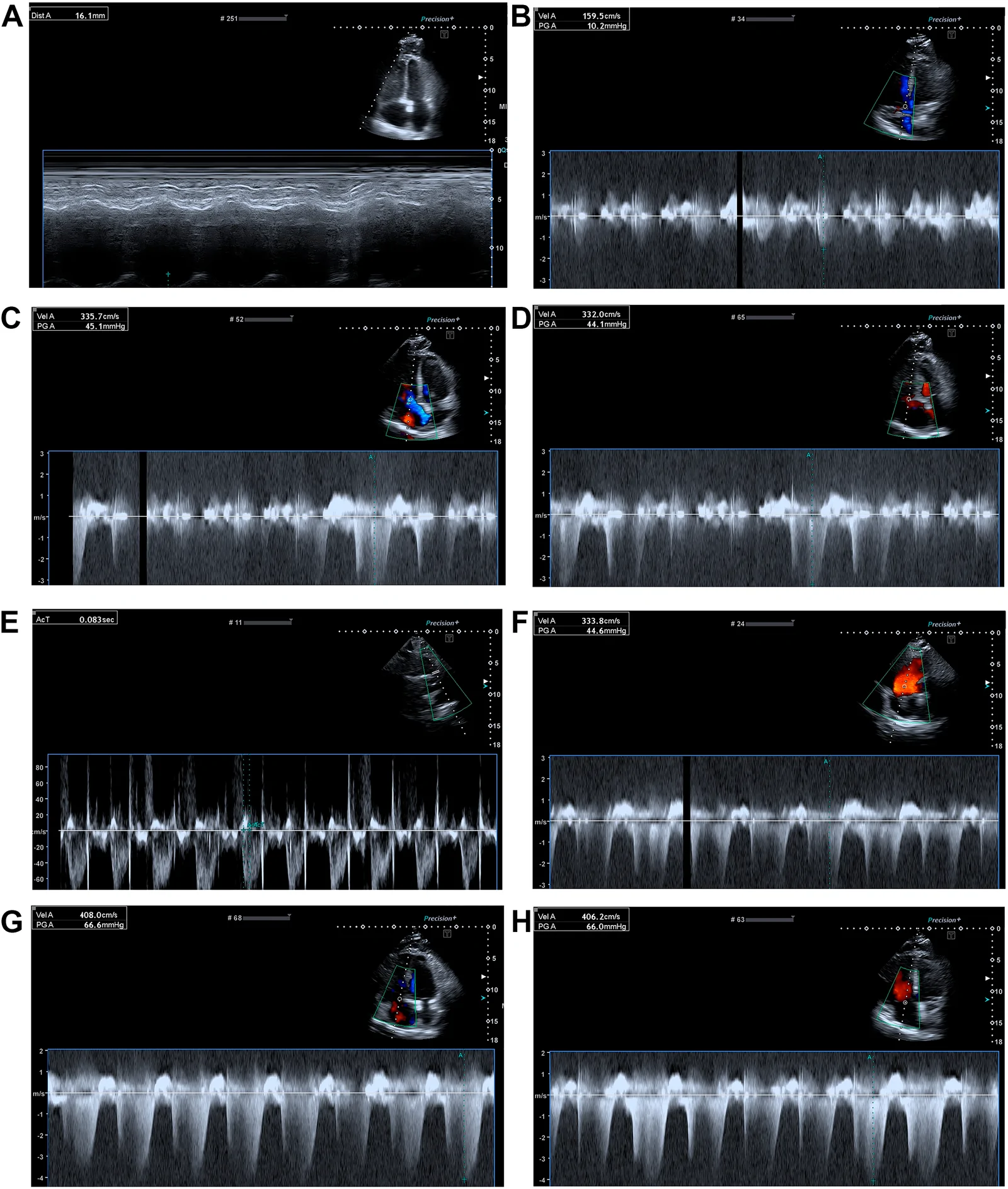
Transthoracic Echocardiography Demonstrating Right-Sided Cardiac Dysfunction and Pulmonary Pressure Overload (A) Apical 4-chamber view demonstrating marked right ventricular and right atrial dilatation with relative compression of the left ventricle, consistent with right ventricular pressure and volume overload. Interventricular septal flattening is noted, suggestive of increased right ventricular pressure. (B to D) Color Doppler imaging across the tricuspid valve demonstrates moderate to severe tricuspid regurgitation. Continuous-wave Doppler interrogation reveals a high-velocity regurgitant jet, indicating elevated right ventricular systolic pressure. (E) Pulsed-wave Doppler assessment of right ventricular outflow tract flow, demonstrating an altered systolic flow pattern consistent with findings suggestive of pulmonary hypertension. (F to H) Continuous-wave Doppler recordings showing increased tricuspid regurgitant jet velocity, supporting the presence of significant echocardiographic evidence suggestive of elevated pulmonary pressures and right ventricular strain, consistent with right-sided heart failure.

Echocardiography was performed to assess right ventricular function and estimate pulmonary pressures, which showed an ejection fraction of 55%, a dilated right ventricle (43 mm) with depressed right ventricular systolic function (tricuspid annular plane systolic excursion [TAPSE]: 10 mm), and elevated estimated right ventricular systolic pressure (RVSP, 55 mm Hg), as shown in Figure 3.

Right heart catheterization, the gold standard for confirming pulmonary hypertension, was not performed because of unavailability at the treating center. Therefore, pulmonary hypertension was considered “suspected” based on echocardiographic findings, rather than confirmed.

**Figure 4:**
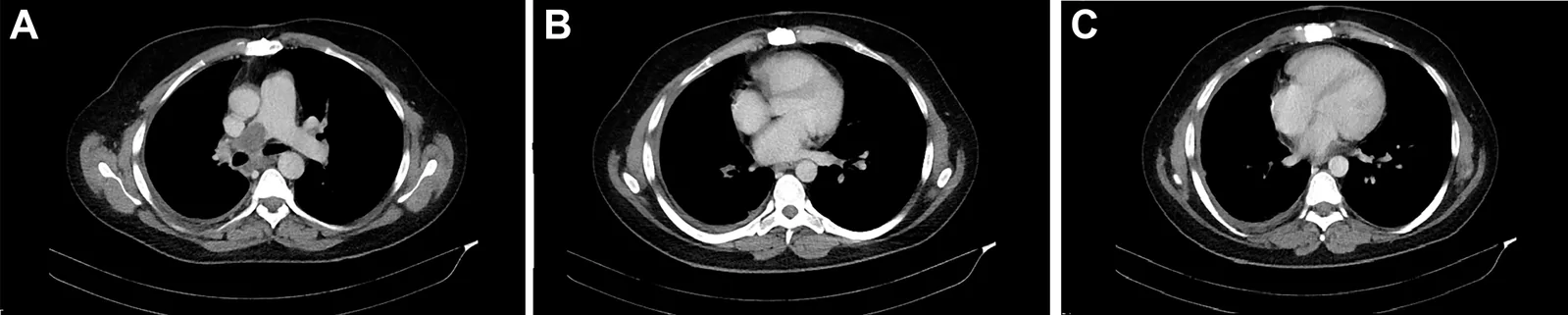
Contrast-Enhanced Computed Tomography Pulmonary Angiography (A) Filling defect within the main pulmonary artery extending into the right pulmonary artery, consistent with acute pulmonary embolism. (B) Complete occlusion of the right pulmonary artery trunk with absence of contrast opacification on the right side, while the left pulmonary artery and its branches demonstrate normal contrast enhancement. (C) Segmental and subsegmental emboli involving the right pulmonary arterial branches, with absence of contrast filling in the right lower lobe branches, whereas preserved contrast opacification is noted in the corresponding left lower lobe branches.

Chest computed tomography (CT) with contrast revealed a large thrombus in the right main pulmonary artery, resulting in complete occlusion. A reduced right lung volume was noted, with a small wedge-shaped area of opacification in the posterior segment of the right upper lung lobe. CT pulmonary angiography showed evidence of a filling defect in the right distal segment of the right main pulmonary trunk extending to mid and lower lobe branches in secondary and tertiary division. Findings were suggestive of submassive PE involving the right lung (Figure 4).

Distinguishing recurrent PE from residual or progressive chronic thromboembolic disease remains challenging in the absence of invasive hemodynamic assessment and perfusion imaging. In this case, the presence of a new intraluminal filling defect favored recurrent embolism. Therefore, the condition was interpreted as recurrent PE.

## Management

The patient was treated with intravenous heparin infusion for 12 hours, followed by systemic thrombolysis with streptokinase infusion and oxygen therapy via concentrator. Systemic thrombolysis was administered despite hemodynamic stability given the presence of severe right ventricular dysfunction, extensive clot burden, hypoxemia, and concern for impending hemodynamic decompensation. In the absence of catheter-directed therapies, systemic thrombolysis was considered the most feasible option in this resource-limited setting.

Cardiothoracic surgical evaluation concluded that although the PE was potentially surgically correctable, repeat pulmonary endarterectomy posed a prohibitive risk given significant right ventricular dysfunction. It was anticipated that the patient would not be able to be weaned from cardiopulmonary bypass without right ventricular assist device support, which was not available locally. Consequently, repeat surgery was deferred. The patient and family were counseled regarding the potential benefits of surgery at specialized international centers with access to advanced mechanical circulatory support. An inferior vena cava (IVC) filter was placed after multidisciplinary discussion because the patient experienced recurrent thromboembolic disease while receiving rivaroxaban and required systemic thrombolysis in the setting of extensive clot burden and proximal lower extremity thrombosis.^1^ The decision was individualized, given the perceived high short-term risk of recurrent embolization and limited availability of advanced rescue therapies. A retrieval strategy was planned after stabilization and establishment of therapeutic anticoagulation. Given the recurrence of PE while on rivaroxaban, anticoagulation was transitioned to warfarin because of suspected anticoagulation failure in this patient. Warfarin was preferred, as it allows therapeutic monitoring via international normalized ratio (INR) and remains the standard of care in recurrent PE in many expert centers. The patient was discharged on optimized medical therapy (warfarin and riociguat 0.5 mg 3 times daily), spironolactone, neurobion, and folic acid.

## Outcome and Follow-Up

**Figure 5:**
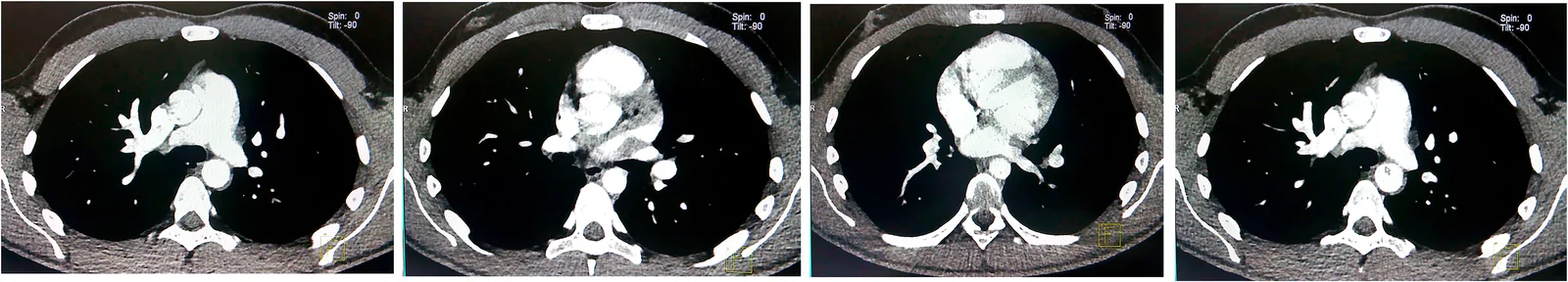
Three-Month Follow-Up Computed Tomography Pulmonary Angiography Demonstrating Resolution of Pulmonary Embolism Follow-up contrast-enhanced computed tomography pulmonary angiography showing marked resolution of previously identified pulmonary arterial thromboembolic filling defects, with improved contrast opacification of the pulmonary arterial branches and restoration of pulmonary blood flow after treatment. These findings are consistent with recovery from pulmonary embolism.

The patient was discharged on oral anticoagulation with warfarin, with regular INR monitoring, along with riociguat therapy and vitamin supplementation. At the 3-month follow-up, the patient demonstrated improvement in functional status from NYHA functional class III to class II, with oxygen saturation improving to 94% on room air. Repeat echocardiography demonstrated partial recovery of right ventricular size and systolic function, with the right ventricular diameter decreasing from 43 to 39 mm, TAPSE improving from 10 to 14 mm, and estimated RVSP decreasing from 55 to approximately 48 mm Hg. The INR remained within the therapeutic range (2.0-3.0). No recurrent thromboembolic events were reported during follow-up. The timeline of clinical events is presented in Table 2. Follow-up CT pulmonary angiography demonstrated resolution of PE, shown in Figure 5.

**Table 2:** Timeline of Clinical Events

Time	Event
5 y prior	Pulmonary endarterectomy performed
Long-term	Rivaroxaban 20 mg daily
1 wk before admission	Progressive dyspnea, SOB
Admission	Hypoxemia + CT-confirmed thrombus
Hospital day 1	Intravenous heparin initiated
Hospital day 2	Streptokinase thrombolysis
Hospital day 3	Surgical evaluation deferred
Hospital day 4	IVC filter placement
Before discharge	Transition to warfarin + riociguat
3 mo follow-up	Functional improvement

IVC = inferior vena cava; SOB = shortness of breath.

## Discussion

Pulmonary embolism is characterized by pulmonary arterial obstruction, which is resolved with adequate anticoagulation; incomplete thrombus resolution can lead to progressive vascular remodeling, fibrosis, and chronic obstruction of pulmonary arteries. Pulmonary endarterectomy remains the definitive treatment for eligible patients.^1^^,^^2^

This case describes recurrent PE in a patient with prior pulmonary endarterectomy and hyperhomocysteinemia who presented with significant right ventricular dysfunction despite rivaroxaban therapy. Although residual chronic thromboembolic pulmonary vascular disease was clinically suspected, definitive confirmation of chronic thromboembolic pulmonary hypertension (CTEPH) could not be established because right heart catheterization was unavailable. Therefore, the diagnostic framework of this report is centered on recurrent PE.

A key challenge in this case was right ventricular dysfunction, which is a major determinant of surgical outcomes and may limit the feasibility of repeat surgical intervention.^3^, ^4^, ^5^ However, therapeutic decisions were guided by a pragmatic multidisciplinary assessment of recurrent PE, worsening right ventricular dysfunction, and limited local treatment options. Anticoagulation (heparin) infusion and systemic thrombolysis (streptokinase) were administered despite the absence of overt hemodynamic instability because of extensive thrombus burden, severe hypoxemia, and significant right ventricular dysfunction, which collectively raised concern for impending hemodynamic deterioration. In the absence of catheter-directed therapies or advanced cardiopulmonary support, systemic thrombolysis was considered the most feasible treatment strategy in this resource-limited setting. Secondly, an IVC filter was placed after a multidisciplinary discussion. The decision was individualized because the patient experienced recurrent thromboembolic disease during anticoagulation therapy and required systemic thrombolysis in the setting of extensive clot burden. Additionally, regarding recurrent PE during rivaroxaban treatment, although direct oral anticoagulants are widely used for venous thromboembolism, their role in chronic thromboembolic disease remains less well established. Current expert consensus and observational data suggest that vitamin K antagonists (eg, warfarin) remain the preferred anticoagulant, particularly in cases of recurrent thrombosis during rivaroxaban therapy.^3^, ^4^, ^5^ A retrieval strategy was planned after stabilization and establishment of therapeutic anticoagulation.

The CHEST-1 trial demonstrated improved exercise capacity and pulmonary hemodynamics with riociguat in patients with inoperable or persistent CTEPH confirmed by right heart catheterization.^6^ Riociguat therapy in this patient was initiated empirically as off-label use because of persistent symptoms, echocardiographic findings suggestive of elevated pulmonary pressures, and significant right ventricular dysfunction. Although definitive diagnosis of CTEPH requires right heart catheterization demonstrating mean pulmonary arterial pressure ≥20 mm Hg, pulmonary arterial wedge pressure ≤15 mm Hg, and pulmonary vascular resistance >2 WU, invasive hemodynamic assessment was unavailable at our institution.

Balloon pulmonary angioplasty is an effective interventional option for inoperable CTEPH,^7^ improving hemodynamics and functional status; however, it was not available in our center, underscoring challenges in resource-limited settings. The patient's severe hyperhomocysteinemia likely contributed to recurrent thrombotic risk despite vitamin therapy. This case highlights key challenges, including the differentiation between recurrent acute PE during rivaroxaban therapy and limited access to advanced therapies. Current guidelines advocate a multimodal, individualized approach that incorporates surgical, interventional, and medical strategies, based on disease severity and resource availability.

Interpretation of the patient's clinical improvement requires caution because several therapeutic interventions were implemented simultaneously, including escalation of anticoagulation, systemic thrombolysis, placement of an IVC filter, oxygen therapy, and initiation of empiric riociguat. Consequently, it is not exactly possible to determine which individual intervention contributed most substantially to the observed short-term improvement. The patient's stabilization most likely reflected the transition of anticoagulation. Severe hyperhomocysteinemia likely contributed to the patient's recurrent thrombotic risk despite folate and vitamin supplementation, as elevated homocysteine levels are recognized risk factors for both arterial and venous thrombosis.

This case highlights several important clinical challenges, including diagnostic uncertainty after prior pulmonary endarterectomy, management of recurrent PE during rivaroxaban therapy, limited access to advanced surgical and interventional therapies, and the need for individualized multidisciplinary decision-making in resource-constrained environments.

## Limitations

A major limitation of this case is the absence of right heart catheterization, which is the gold standard for confirming CTEPH. This limits the ability to make a definitive diagnostic classification and to perform hemodynamic assessment. Additionally, this report represents a single patient's experience; therefore, the findings do not establish a causal relationship between suspected anticoagulant failure and recurrent PE. Furthermore, long-term clinical outcomes after anticoagulation therapy switch and IVC filter placement were not available at the time of reporting.

## Conclusions

This case highlights the complexity of managing recurrent PE after pulmonary endarterectomy in a resource-limited setting, particularly in patients with persistent thrombotic risk factors such as hyperhomocysteinemia. The report underscores the importance of maintaining diagnostic caution when invasive hemodynamic confirmation is unavailable and demonstrates the role of individualized multidisciplinary decision-making when advanced surgical and interventional therapies are inaccessible.

## Funding Support and Author Disclosures

The authors have reported that they have no relationships relevant to the contents of this paper to disclose.

**Take Home Messages**

• Recurrent pulmonary embolism may occur despite anticoagulation with direct oral anticoagulants such as rivaroxaban, particularly in patients with hyperhomocysteinemia.
• Patients with hyperhomocysteinemia remain at increased risk for both arterial and venous thrombosis despite homocysteine-lowering therapy with folic acid and vitamin B supplementation.
• An individualized multimodal strategy, including transition to warfarin and temporary IVC filter placement, may be considered in selected high-risk patients when advanced surgical or interventional therapies are unavailable.

## References

[1] Kim N.H., D'Armini A.M., Delcroix M. (2024). Chronic thromboembolic pulmonary disease. Eur Respir J.

[2] Delcroix M., Godinas L., Quarck R., Wagner T.O.F., Humbert M., Wijsenbeek M. (2023). *Rare Diseases of the Respiratory System* (ERS Monograph).

[3] Jenkins D.P., Martinez G., Salaunkey K., Reddy S.A., Pepke-Zaba J. (2023). Perioperative management in pulmonary endarterectomy. Semin Respir Crit Care Med.

[4] Reddy S.A., Swietlik E.M., Robertson L. (2023). Natural history of chronic thromboembolic pulmonary disease with no or mild pulmonary hypertension. J Heart Lung Transplant.

[5] Astashchanka A., Kerr K.M., Yang J.Z. (2023). Repeat pulmonary thromboendarterectomy outcomes: a 15-year single-center retrospective review. J Thorac Cardiovasc Surg.

[6] Ghofrani H.A., D'Armini A.M., Grimminger F. (2013). Riociguat for the treatment of chronic thromboembolic pulmonary hypertension. N Engl J Med.

[7] Lang I.M., Andreassen A.K., Andersen A. (2023). Balloon pulmonary angioplasty for chronic thromboembolic pulmonary hypertension: a clinical consensus statement of the ESC working group on pulmonary circulation and right ventricular function. Eur Heart J.

